# Reply: Microvascular Dysfunction and Residual Angina After PCI

**DOI:** 10.1016/j.jacadv.2026.103373

**Published:** 2026-10-22

**Authors:** Frédéric Bouisset, Kazumasa Ikeda, Takuya Mizukami, Carlos Collet

**Affiliations:** a Department of Cardiology, Toulouse University Hospital, France; b Department of Cardiology, Tokyo Medical University Hachioji Medical Center, Tokyo, Japan; c Division of Clinical Pharmacology, Department of Pharmacology, Showa University, Tokyo, Japan; d Clinical Trial Center, Cardiovascular Research Foundation, New York, New York, USA

We thank Dr Dziewierz and colleagues for highlighting the distinction between attained health status at follow-up and symptomatic improvement. We agree that baseline symptom burden and the ceiling of the Seattle Angina Questionnaire angina frequency (SAQ-AF) scale complicate interpretation of unadjusted comparisons. In our study,^1^ patients with focal coronary artery disease and concomitant coronary microvascular dysfunction (CMD) had lower SAQ-AF scores at baseline and 12 months. However, the mean improvement after percutaneous coronary intervention (PCI) did not differ significantly between patients with and without CMD (22.68 vs 18.64 points; *P* = 0.350).

To address the baseline imbalance, we fitted a linear regression model of 12-month SAQ-AF incorporating baseline SAQ-AF, age, sex, pre-PCI microvascular resistance reserve (MRR), and pull back pressure gradient (PPG). The analysis included 201 patients with complete data. The MRR × PPG interaction was significant (coefficient 7.00; 95% CI: 1.88-12.1; *P* = 0.008), indicating that the association between pre-PCI MRR and follow-up SAQ-AF varied according to epicardial disease pattern. This is consistent with the greater residual angina observed in patients with focal coronary artery disease and concomitant CMD. This finding concerns attained health status at 12 months and does not establish modification of the symptomatic benefit attributable to PCI.

We also agree that a single pre-PCI measurement cannot establish persistent CMD. Among 151 patients with paired measurements, 35 had MRR <3.0 at both assessments, 20 crossed from <3.0 to ≥3.0, 29 crossed in the opposite direction, and 67 remained at ≥3.0. CMD prevalence was 36.4% before and 42.4% after PCI. Although MRR accounts for epicardial disease and driving pressures,^2^ physiological changes, regression to the mean, and variability of bolus thermodilution^3^ may contribute to reclassification. Twenty patients (13.2% of those with paired measurements) crossed above the diagnostic threshold. These observations do not establish resolution of CMD by revascularization and should not be interpreted as a resolution of CMD by revascularization.^3^

To assess the relationship between postprocedural microvascular function and subsequent symptoms, we fitted a separate model incorporating post-PCI MRR. Among the 151 patients with post-PCI measurements, MRR was not significantly associated with 12-month SAQ-AF (coefficient, −0.12; 95% CI: −0.82-0.59; *P* = 0.743). This analysis therefore does not support an overall association between post-PCI MRR and residual angina.

PCI targets epicardial resistance rather than microvascular dysfunction. Patients with concomitant CMD may therefore experience symptomatic improvement through relief of epicardial obstruction while continuing to have residual symptoms. Our findings suggest that the relationship between pre-PCI microvascular function and 1-year health status varies with epicardial disease pattern defined by PPG. They do not establish reduced symptomatic benefit from PCI in patients with CMD. Given the observational design and modest sample size, these findings remain hypothesis-generating.
